# Biomarker panels for characterizing microbial community biofilm formation as composite molecular process

**DOI:** 10.1371/journal.pone.0202032

**Published:** 2018-08-09

**Authors:** Magnus Bosse, Alexander Heuwieser, Andreas Heinzel, Arno Lukas, Guilherme Oliveira, Bernd Mayer

**Affiliations:** 1 Emergentec Biodevelopment GmbH, Vienna, Austria; 2 Vale Institute of Technology, Belém, Pará, Brazil; University of British Columbia, CANADA

## Abstract

Microbial consortia execute collaborative molecular processes with contributions from individual species, on such basis enabling optimized molecular function. Such collaboration and synergies benefit metabolic flux specifically in extreme environmental conditions as seen in acid mine drainage, with biofilms as relevant microenvironment. However, knowledge about community species composition is not sufficient for deducing presence and efficiency of composite molecular function. For this task molecular resolution of the consortium interactome is to be retrieved, with molecular biomarkers particularly suited for characterizing composite molecular processes involved in biofilm formation and maintenance. A microbial species set identified in 18 copper environmental sites provides a data matrix for deriving a cross-species molecular process model of biofilm formation composed of 191 protein coding genes contributed from 25 microbial species. Computing degree and stress centrality of biofilm molecular process nodes allows selection of network hubs and central connectors, with the top ranking molecular features proposed as biomarker candidates for characterizing biofilm homeostasis. Functional classes represented in the biomarker panel include quorum sensing, chemotaxis, motility and extracellular polysaccharide biosynthesis, complemented by chaperones. Abundance of biomarker candidates identified in experimental data sets monitoring different biofilm conditions provides evidence for the selected biomarkers as sensitive and specific molecular process proxies for capturing biofilm microenvironments. Topological criteria of process networks covering an aggregate function of interest support the selection of biomarker candidates independent of specific community species composition. Such panels promise efficient screening of environmental samples for presence of microbial community composite molecular function.

## Introduction

Microbial communities display cooperation and leverage on synergies, all embedded on the level of entangled molecular processes across individual species. Occurrence of such traits is tightly controlled via given species genotypes and environmental conditions, and centers on cost and benefit [1]. Extreme habitats including acid mine drainage (AMD), saline lakes or hot springs are particularly prone to formation of cooperative microbial communities.

Cooperation displays as higher order function, i.e. molecular processes executed by the community being not reducible to any of its individual species alone. Higher order molecular functions may alter local environmental parameters, in turn imposing a feedback on cooperation mechanisms, individual species abundance and specific involvement. Examples for such community properties include occupation of sites lacking viable conditions for individual species, division of labor, and fostering significant increase in metabolic flux [2].

Microbial communities hold profound relevance in industrial processes. One example is bioleaching, i.e. extraction of base metals from sulfide ores [3]. A second application area is bioremediation, offering alternatives to abiotic neutralization processes [4]. Optimization of bio-based processes needs to addresses eminent efficiency constraints as well as process resilience issues. Challenges include design of suitable consortia for sufficient process efficiency, de-novo seeding of bioleaching or bioremediation operations, optimization of pre-existing community compositions through addition of further members, or abiotic interference for adjusting environmental conditions [5, 6]. Any rational design of consortia or selective interference with existing communities requires an understanding of community aggregate molecular function and respective phenotypic properties, not necessarily deducible from a listing of a community’s species composition.

Rational design and optimization of community function was recently demonstrated for copper bioleaching [7]. However, metagenomics of environmental samples identified broad species diversity. To date, about a dozen studies were conducted in both AMD environments and bioleaching operations focusing on inventorying metabolic and genetic diversity of acidophile communities.

Extrapolation from a species inventory to capabilities of embedded community molecular function is limited, as resolution of species interaction on a molecular function level is needed. Next to allowing optimization of given consortia properties such knowledge promises particular utility in molecular function prospecting. Such scenario focuses on identification of communities in environmental samples exhibiting specific aggregate molecular function of interest [8].

Recalling that increased efficiency in bioleaching resembles a composite molecular process in the sense of a microbial community aggregate function, a relevant proxy for such molecular configuration is given as molecular biomarkers. Use of molecular biomarkers is further supported by recently identified evidence that function and underlying molecular processes of a community may be preserved while the species composition of a community exhibits a dynamic flux [9]. Further, communities collected at different sites may differ in species composition while being functionally comparable [10].

Biomarker identification approaches include explorative methods, involving comprehensive functional gene arrays as well as various omics tools. For example, Roume et al. integrated omics profiling results on molecular interaction networks for characterizing the impact of environmental conditions on microbial communities relevant in biological waste water treatment [11]. On the basis of metagenomics, -transcriptomics and -proteomics a metabolic network was derived. Abundance levels from omics were combined with network characteristics for identifying relevant molecular function assignable to selected environmental conditions. Kuang et al. reported a comparative analysis of taxonomy-based and functional gene-based characterization of natural AMD microbial assemblages, indicating superiority of molecular parameters for predicting microbial community characteristics [12].

Analysis of molecular context is of particular value when aiming at characterizing composite function. Microbial community representation has to focus on the species set **M** composed of **n** individual microbial species **m**^**i**^, **m**^**j**^, … **m**^**n**^ which together constitute the community. A higher order function results out of a composite molecular process **p**^**i**^, principally grounded on the set of protein coding genes **g**^**i**^, **g**^**j**^ of all community species, hence the community genome **G**.

The composite molecular process sees involvement of molecular functionality (gene products) from different species, needing modeling of the microbial community on the level of **G** for deriving the specific set of cross-species processes **p**^**i**^, **p**^**j**^ responsible for triggering phenotypic community characteristics. A biomarker in its definition serves as proxy for the status of a molecular process. Having identified candidate processes **p**^**i**^, **p**^**j**^ responsible for a molecular function of interest in turn allows selecting corresponding candidate biomarkers **b**^**i**^, **b**^**j**^, where **b**^**i**^ monitors the state of process **p**^**i**^, and **b**^**j**^ of **p**^**j**^, respectively. Recalling the finding of community stability in terms of composite function but not in terms of species composition, such a biomarker panel may offer generalization in prospecting of environmental samples. Further, such biomarker panel approach realized as multiplexed assay may prove efficient for high throughput analysis of environmental samples.

On this basis we propose an *in silico* workflow for identifying biomarker candidate panels aimed at reflecting aggregate microbial community properties. Starting with a reference inventory of a functionally relevant subset of **G** further combined with interaction information allows deriving a molecular process graph. On the graph level, topological parameters aiming at resembling molecular process proxies as well as species coverage considerations are used for biomarker candidate selection. The resulting candidate biomarker panel is thus specific for the function in focus but to some degree agnostic regarding actual species composition. Such candidate panel promises generic applicability in bioprospecting regarding a composite molecular function of interest.

While the workflow is applicable for studying various instances of microbial community aggregate function we in the following focus on community cooperation in forming a biofilm microenvironment in copper bioleaching/AMD. Biofilms can be instantiated by certain individual microbial species, however, in most environmental settings multiple species contribute molecular function to this microenvironment. In selected scenarios such environment provides grounds for emergent community phenotypes, including significant increase in bioleaching efficiency or initiation of AMD [13–15].

## Materials and methods

**Fig 1:**
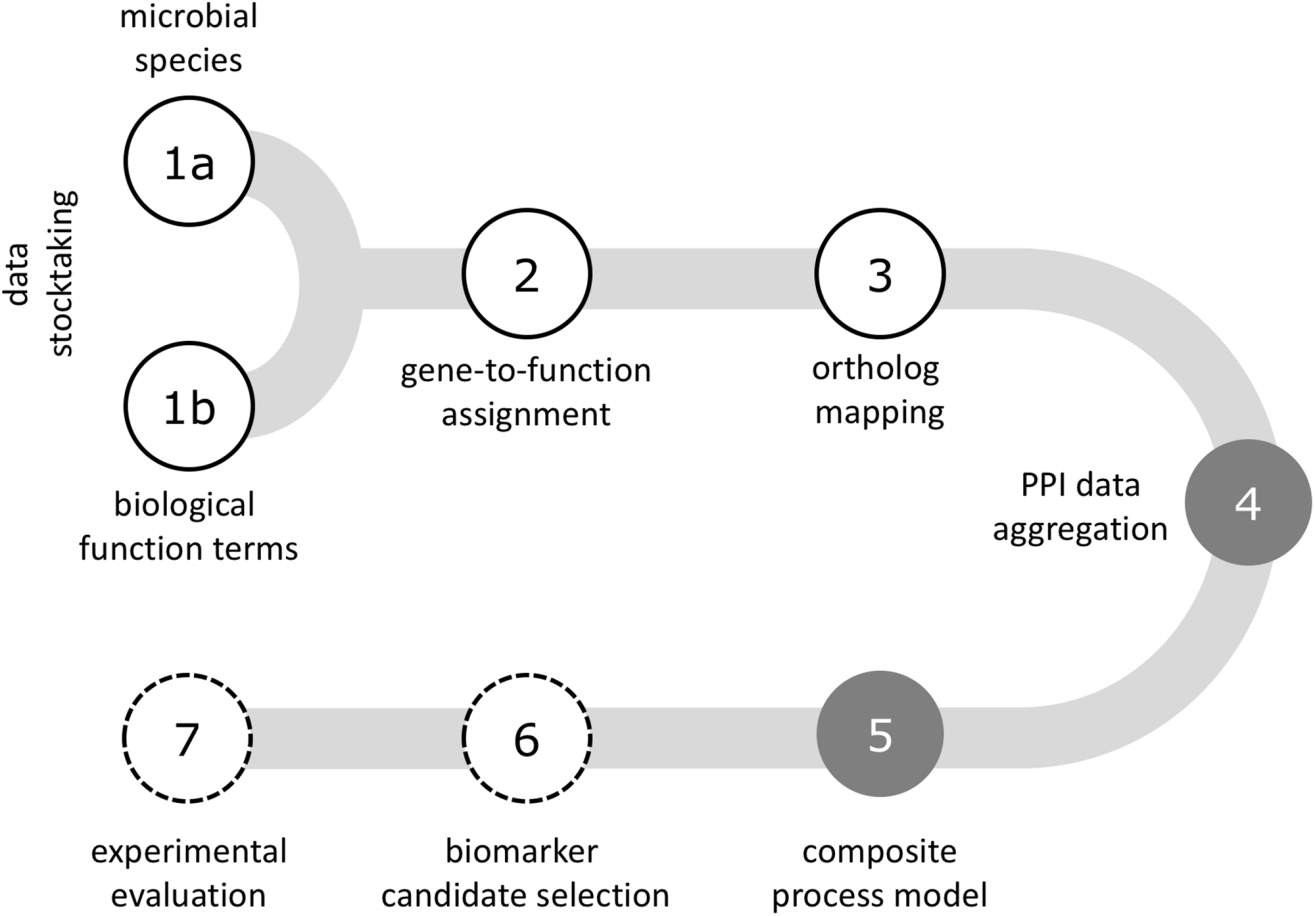
Workflow for candidate biomarker selection. Microbial species and molecular feature details: step 1—data stocktaking at the microbial species and biological function level; step 2—assignment of genes to biological function terms. Interaction data and networks: step 3—all-against-all ortholog mapping; step 4—aggregation of protein interaction data; step 5—deriving a molecular process model. Biomarker candidates and experimental evaluation: step 6—selection of biomarker candidates utilizing network topology characteristics; step 7—experimental evaluation of biomarker candidates.

The workflow illustrated in Fig 1 aligns data retrieval, integration and mapping on a protein interaction network, followed by utilizing topology characteristics of a graph representing aggregate function for candidate biomarker selection, complemented by experimental evaluation.

In a first step scientific literature is screened for microbial communities reported as relevant in a given focus (as copper bioleaching/AMD) for cataloguing constituent species genomes. In addition, biological community aggregate molecular functions reported as relevant are extracted. In a second step, catalogued genes are assigned to community molecular functions. Third, an all-against-all ortholog map is created for the gene set assigned to the biological function term in focus (e.g. biofilm formation), followed by consolidating protein-protein interaction information for the set of orthologs (step 4). With a gene set and respective interaction data at hand a molecular process model approximating the function term is retrieved (step 5), on this basis deriving topological parameters of the interaction network for guiding biomarker candidate selection (step 6). Finally, candidate biomarkers are forwarded to experimental evaluation (step 7).

### Microbial species and molecular feature details

A list of natural acid mine drainage sites, major industrial bioleaching operations as well as suitable laboratory scale experiments, in the following referred to as ‘sites’, was compiled by mining the National Library of Medicine (NCBI) PubMed scientific literature repository in publication title and abstract for the terms ‘bioleaching’ and ‘acid mine drainage’. For all identified sites respective mineral endowment was extracted. For industrial mining sites additional mineral composition data were sourced from the Mindat database [16]. Altogether 70 sites were identified, among these being 18 copper sites (either AMD associated with copper mineral or an industrial copper plant, Table 1). Site-specific scientific references are provided in S1 Table.

**Table 1:** Listing of copper sites.

site	mineral type	mineral	site	mineral type	mineral
Aguablanca Mine (Spain)	Ni/Cu	copper residues	Lechang Mine (China)	Pb/Zn/Cu/Cd	mixed sulphides with contents of malachite
Cantareras Mine (Spain)	Cu	copper sulfides	Mynydd Parys Mine (UK)	Cu/Pb/Zn	chalcocite, chalcopyrite, covellite
El Abra Mine (Chile)	Cu	chalcocite, chalcopyrite, covellite	Olavsgruva Mine (Norway)	Cu	chalcopyrite, cubanite
Escondida Mine (Chile)	Cu	chalcocite, chalcopyrite, covellite	Richmond Mine (USA)	Cu	chalcopyrite
Spence Leach Plant (Chile)	Cu	chalcocite, chalcopyrite, covellite	Saão Domingos Mine (Portugal)	Cu	chalcanthite, chalcopyrite, cupriferous pyrite, malachite
Fankou Mine (China)	Pb/Zn	bournonite, chalcopyrite, tetrahedrite	Selebi-Phikwe Mine (Botswana)	Cu/Ni	chalcopyrite, malachite
Freiberg Mine (Germany)	Au/Cu	freibergite	Tong Shankou Mine (China)	Cu/Mo	chalcocite, chalcopyrite
Kristineberg Mine (Sweden)	Au/Ag/Cu/Zn	mixed sulphides with contents of chalcopyrite	Yinshan Mine (China)	Pb/Zn/Cu/Ag/Au	porphyry copper reserve (chalcocite, chalcopyrite, covellite, freibergite, tetrahedrite)
La Andina Mine (Chile)	Cu	porphyry copper tailing (bornite, chalcocite, chalcopyrite, covellite)	Yongping Mine (China)	Cu	porphyry copper reserve (bornite, chalcopyrite, malachite, tetrahedrite)

Copper site details including mining site name and geographical area, complemented by details on specific mineral type and copper mineral composition.

Next, scientific literature covering the sites was screened for inventorying involved microbial species. 133 microbial species could be retrieved, of which 42 are specifically associated with one or more of the 18 copper sites.

For each of the copper site-associated microbial species gene-centric annotation was retrieved from public databases as described previously [17]. Briefly, species as well as protein coding gene identifiers were retrieved from the Pathosystems Resource Integration Center (PATRIC) [18], the Integrated Microbial Genomes database (IMG) [19], as well as Universal Protein Resource (UniProt) [20], with the UniProt identifier (ID) used henceforth as unique sequence identifier.

Each copper site-associated species was evaluated for availability of annotation data for assigning protein coding genes to functional groups (COG) [21], and for presence of protein interaction information in the Search Tool for the Retrieval of Interacting Genes/Proteins (STRING) [22]. For 25 of the 42 species COG-annotated proteomes, and for 14 species protein interaction data could be retrieved. The resulting inventory of copper site-associated species as well as their respective annotation is provided in Table 2. Species-specific scientific references with respect to AMD and bioleaching sites are provided in S1 Table.

**Table 2:** Microbial species annotation details.

species	domain	# sites	COG	STRING
*Acidimicrobium ferrooxidans* DSM 10331	B	2	y	y
*Acidiphilium acidophilum* ATCC 27807	B	3		
*Acidiphilium cryptum* JF-5	B	4	y	y
*Acidiphilium rubrum* ATCC 35905	B	1		
*Acidiphilium* strain SJH	B	1		
*Acidithiobacillus albertensis strain* DSM 14366	B	1		
*Acidithiobacillus caldus* ATCC 51756	B	3	y	y
*Acidithiobacillus ferrivorans* SS3	B	4	y	y
*Acidithiobacillus ferrooxidans* ATCC 23270	B	11	y	y
*Acidithiobacillus ferrooxidans* ATCC 53993	B	1	y	y
*Acidithiobacillus thiooxidans* ATCC 19377	B	4		
*Acidobacterium capsulatum* ATCC 51196	B	3	y	y
*Acidobacterium strain* Thars1	B	1		
*Acidocella aromatica* strain PFBC	B	1		
*Acidocella facilis* ATCC 35904	B	2	y	
*Acidovorax* sp. JS42	B	1	y	y
*Alicyclobacillus acidocaldarius* DSM 446	B	2	y	y
*Alicyclobacillus disulfidooxidans*	B	1		
*Alicyclobacillus pomorum* DSM 14955	B	1	y	
*Candidatus Micrarchaeum acidiphilum* ARMAN-2	A	2		
*Candidatus Parvarchaeum acidiphilum* ARMAN-4	A	2	y	
*Candidatus Parvarchaeum acidophilus* ARMAN-5	A	2	y	
*Ferrimicrobium acidiphilum* DSM 19497	A	1	y	
*Ferroplasma acidarmanus* fer1	A	2	y	y
*Ferroplasma acidiphilum*	A	2		
*Ferroplasma cupricumulans*	A	1		
*Ferrovum myxofaciens* P3G	B	4	y	
*Leptospirillum ferriphilum* DSM 14647	B	6	y	
*Leptospirillum ferriphilum* ML-04	B	1	y	
*Leptospirillum ferriphilum* Sp-Cl	B	1		
*Leptospirillum ferrodiazotrophum*	B	1		
*Leptospirillum* sp. Group IV UBA	B	3	y	
*Leptospirillum ferrooxidans* C2-3	B	9	y	
*Leptospirillum rubarum* (group II)	B	2		
*Picrophilus torridus* DSM 9790	A	3	y	y
*Sulfobacillus Benefaciens*	B	1		
*Sulfobacillus thermosulfidooxidans* str. Cutipay	B	2	y	
*Sulfobacillus thermotolerans*	B	2		
*Sulfolobus acidocaldarius* DSM 639	A	1	y	y
*Thermoplasma acidophilum* DSM 1728	A	1	y	Y
*Thiobacillus prosperus* DSM 5130	B	1		
*Thiomonas arsenitoxydans* 3As	B	1	y	Y

Given is the microbial species name and domain (A: Archaea; B: Bacteria), and number of mining sites with positive identification of a given species, complemented with molecular annotation coverage on the level of COG (“y”) and availability of interaction information from STRING (“y”).

A matrix of biological function terms and genes of relevance in copper bioleaching was obtained by manual data extraction from scientific publications reporting species given in Table 2. Further, molecular features explicitly discussed in relation to AMD or bioleaching (being either investigated in reductionist approaches or identified as relevant in omics profiling) were extracted and recorded together with respective COG assignment and functional context. Clusters of functional terms based on relative COG term endowments were identified from the data matrix using hierarchical clustering as provided in R 3.1.2 [23].

### Interaction data and networks

A comprehensive all-against-all ortholog screening of the 25 species holding COG annotation was executed using the InParanoid pipeline (version 4.1) with default settings [24]. As input files protein sequence data from the PATRIC database were used. Sequence data for two organisms lacking coverage in PATRIC (*Leptospirillum* ssp. Group IV ‘UBA BS’ and *T*. *arsenitoxydans*) were sourced from the NCBI RefSeq database.

Protein interaction information was retrieved from STRING (combined score, database version 10.0a) and subsequently aggregated across all 25 species using ortholog screening results to obtain an interactome on the ortholog level. This procedure allowed inclusion of protein coding genes of species as such not represented in the STRING database on the level of identified ortholog sequences from respective species being covered in STRING. The resulting interaction matrix was used to derive an ortholog network.

### Biomarker candidates and experimental evaluation

Topological parameters of the ortholog graph were calculated using the NetworkAnalyzer application of Cytoscape version 3.2.1 [25], network visualization was done in Gephi version 0.8.2 [26]. Correlation analysis, logistic regression analysis and significance testing (two-sided t-test) among graph measures, species coverage and candidate biomarker abundance retrieved from experimental reference experiments were performed in R 3.1.2.

For evaluation of candidate biomarkers selected on the ortholog graph level resembling the composite process of biofilm formation available omics profiles were used. A scientific literature review identified omics profiles in scope of bioleaching/AMD providing a resolution on the level of protein coding gene products. Data sets of specific interest needed to resemble meta-transcriptomics or -proteomics profiles covering microbial communities exhibiting biofilms in various environmental or laboratory conditions.

Two data sets appeared particularly suitable with respect to biofilm formation serving as surrogate of bioleaching/AMD. The first study conducted by Belnap et al. covers proteomics profiling [14]. The authors demonstrate a laboratory adaptation of a natural AMD biofilm sourced from the Richmond Mine (Iron Mountain, USA). The communities are of low species diversity and dominated by *Leptospirillum* and *Ferroplasma* species. To assess biofilm functional traits quantitative proteomics comparison of natural versus laboratory settings was conducted, complemented by determining biomass to estimate *in situ* chemoautotrophic production. Productivity in the laboratory biofilm community was nearly five times lower compared to its natural equivalent, being reflected in differential abundance of a range of metabolic proteins. The authors concluded that the differences in both production as well as protein expression profiles reflect metabolic stress in the laboratory biofilm. After optimization of laboratory culture conditions (particularly NH_3_ supply, as well as KCl, K_2_HPO_4_, MgSO_4_ and CaSO_4_ concentrations), community proteomics profiles resembled the environmental setting particularly in respect to expression of stress response proteins and community growth rates.

For calculation of differences in expression in the proteomics data the respective log2-ratios of abundance when comparing the optimized laboratory culture with a natural biofilm sample were subtracted from the respective abundance values comparing a standard laboratory culture with a natural biofilm. Respective fold changes are therefore indicative for the level of biofilm formation and are used in biomarker candidate evaluation with respect to prospecting the capacity of forming a composite biofilm microenvironment.

A second data source originates from a transcriptomics profiling study on bacterial communities isolated from the Rio Tinto acid mine drainage system (Sierra Morena Mountains, Spain) published by Moreno-Paz et al. [13]. The communities were comprised mainly of *A*. *ferrooxidans* as well as *L*. *ferriphilum* and *L*. *ferrooxidans*, respectively. Further community members worth noting include *Acidimicrobium ferrooxidans* as well as several *Acidiphilum*, *Ferroplasma* and *Sulfobacillus* species, respectively. The study compared transcriptional profiles of natural planktonic and biofilm-associated consortia to gain insights into physiological differences between free and the sessile community fractions. This approach allowed a precise assessment of biofilm physiology determined on a genomic array of *L*. *ferrooxidans*, identifying as main contributors to the biofilm situation genes coding for quorum sensing, motility and chemotaxis, as well as matrix component production and transport.

In biomarker evaluation the fold changes (log2-ratios) are used contrasting planktonic and biofilm status in focus of biofilm formation capacity.

A third study conducted by Christel et al. compared a culture of *L*. *ferriphilum* in continuous vs. bioleaching conditions, the latter being grown on 2% (wt/vol) chalcopyrite [27]. The authors executed the experiments in a stirred tank bioreactor with samples collected from the culture medium rather than directly from the biofilm. A low transcript level of biofilm-associated genes was identified, seeing no significant up-regulation after addition of chalcopyrite for triggering bioleaching.

This study serves as negative control experiment for candidate biomarker evaluation, utilizing fold changes (log2-ratios) comparing cultures in absence and presence of chalcopyrite.

Protein coding genes addressed in the three experimental studies were assigned to the ortholog graph via Basic Local Alignment Search Tool 2 (BLAST2) [28], selecting the top-scoring matches for allowing assignment of abundance values to biofilm network nodes.

## Results and discussion

### Microbial species, molecular features and function terms

An important prerequisite for candidate biomarkers to be used in microbial community screening is applicability in absence of prior information about specific community species composition. Therefore a meta-analysis approach is applied starting with an inventory of microbial communities being involved in either bioleaching or acid mine drainage. Consortia retrieved from 70 environmental sites in focus of bioleaching/AMD provide 133 individual species. 18 sites report on copper sulfite ores with in total 42 individual microbial species. The majority (32) is comprised of Bacteria, complemented by Archaea. The four most prevalent species are *A*. *ferrooxidans* (identified in 11 sites), *L*. *ferrooxidans* (9 sites), *L*. *ferriphilum* (7 sites) *and A*. *cryptum*, *A*. *ferrivorans*, *A*. *thiooxidans* and *F*. *myxofaciens* (4 sites each). Each site further reports a diverse and distinctive array of less frequent species.

Manual literature curation of molecular features involved in molecular processes relevant in acid mine drainage and/or bioleaching together with their molecular functional annotation identifies 14 molecular function categories holding 1,696 protein coding genes (S2 Table). The Clusters of Orthologous Groups (COG) ontology allows a structured overview on the functional complexity of communities. 25 of the 42 species hold annotation in COG terms. Table 3 provides a gene coverage matrix assigning the gene set to molecular function categories, further relating to COG terms. Function category-specific scientific references are provided in S1 Table.

**Table 3:** Molecular function terms and COG term assignment.

COG term	SO	IO	NF	AU	BF	QS	CT	OT	PT	OS	CR	HM	TR	CA
**C** Energy production and conversion														
**D** Cell cycle control and mitosis														
**E** Amino Acid metabolism and transport														
**F** Nucleotide metabolism and transport														
**G** Carbohydrate metabolism and transport														
**H** Coenzyme metabolism														
**I** Lipid metabolism														
**J** Translation														
**K** Transcription														
**L** Replication and repair														
**M** Cell wall/ membrane/ envelope biogenesis														
**N** Cell motility														
**O** Post-translational modification, …														
**P** Inorganic ion transport and metabolism														
**Q** Secondary metabolites biosynthesis, …														
**T** Signal transduction														
**U** Intracellular trafficking and secretion														
**V** Defence mechanisms														
**W** Extracellular structures														
**X** Mobilome: prophages, transposons														

Molecular function terms of relevance in copper bioleaching/AMD assigned to COG categories and the number of genes identified for each of these terms: SO—sulfur oxidation (128); IO—iron oxidation (106); NF—nitrogen fixation (78); AU—ammonia uptake (97); BF—biofilm formation (272); QS—quorum sensing (204); CT—chemotaxis (150); OT—osmo tolerance (26); PT—pH tolerance (106); OS—oxidative stress response (170); CR—copper resistance (117); HM—heavy metal resistance (107); TR—toxicant resistance (100); CA—cold acclimation (35). Shading indicates representation of a COG category in a functional term with respect to assigned molecular features. Highly prevalent COG categories in a given function term are indicated in dark grey.

20 of the 26 COG terms are addressed in the AMD/bioleaching context, with specific function terms seeing varying assignment to COG terms. For instance, chemotaxis is associated with 5 COG terms, on a gene count level mainly involving cell motility (N) and signal transduction (T). Other terms are considerably more complex, specifically the function term biofilm formation involving 19 COG terms. Main contributions come from cell wall/membrane/envelope biogenesis (M), cell motility (N), signal transduction (T), intracellular trafficking and secretion (U) as well as extracellular structures (W). Other complex functions with diverse COG term involvement include quorum sensing together with toxicant and copper resistance. Genes lacking COG annotation or being assigned to the unspecific COG terms R (General Functional Prediction only) and S (Function Unknown) are not further considered.

Related function terms map to similar COG term combinations. For biofilm formation, COG terms include cell wall biogenesis, signal transduction and trafficking/secretion categories. Comparable patterns of COG category involvement are found for quorum sensing and chemotaxis, reflecting close biological relationship.

For deriving a biomarker candidate panel capturing major molecular aspects of relevance in both AMD and copper bioleaching, function terms covering a broad set of COG terms appear preferential, ideally further seeing broad involvement of microbial species. Of the 14 function terms, biofilm formation exhibits particularly broad COG term assignment as well as species coverage across the original catalogue of 25 copper mineral associated microbes, providing all ingredients for establishment of higher order molecular function. Furthermore, from a biological function perspective, establishment of biofilms is relevant in both acid mine drainage as well as bioleaching. As demonstrated, enrichment of consortia with microbes particularly competent in formation of biofilms significantly boosts bioleaching process efficiency [15]. Biofilms resemble macroscopic structures and provide an enabling microenvironment for inhabitant microbes of different species to coordinate their interactions for gaining overall benefit. In the ecological context, formation of biofilms on metal sulfide surfaces serves protective aims under extreme environmental conditions with catalyzing effect in energy supply, subsequently triggering leaching of minerals [29].

Accordingly, the set of genes assigned to the aggregate function of biofilm formation is used as basis for deriving a protein coding gene interaction network, subsequently serving for biomarker panel selection.

### Ortholog interaction network

According to data retrieval and functional category assignment 272 protein coding genes are identified in the molecular context of biofilm formation. Ortholog screening of this set in the genomes of the 25 species provides 3,106 orthologs, identifying at least one ortholog for all but 23 genes. 57 of the 272 genes hold orthology to other members of the biofilm gene set and are removed to avoid biases from multiple sequence representation in subsequent molecular network retrieval.

**Fig 2:**
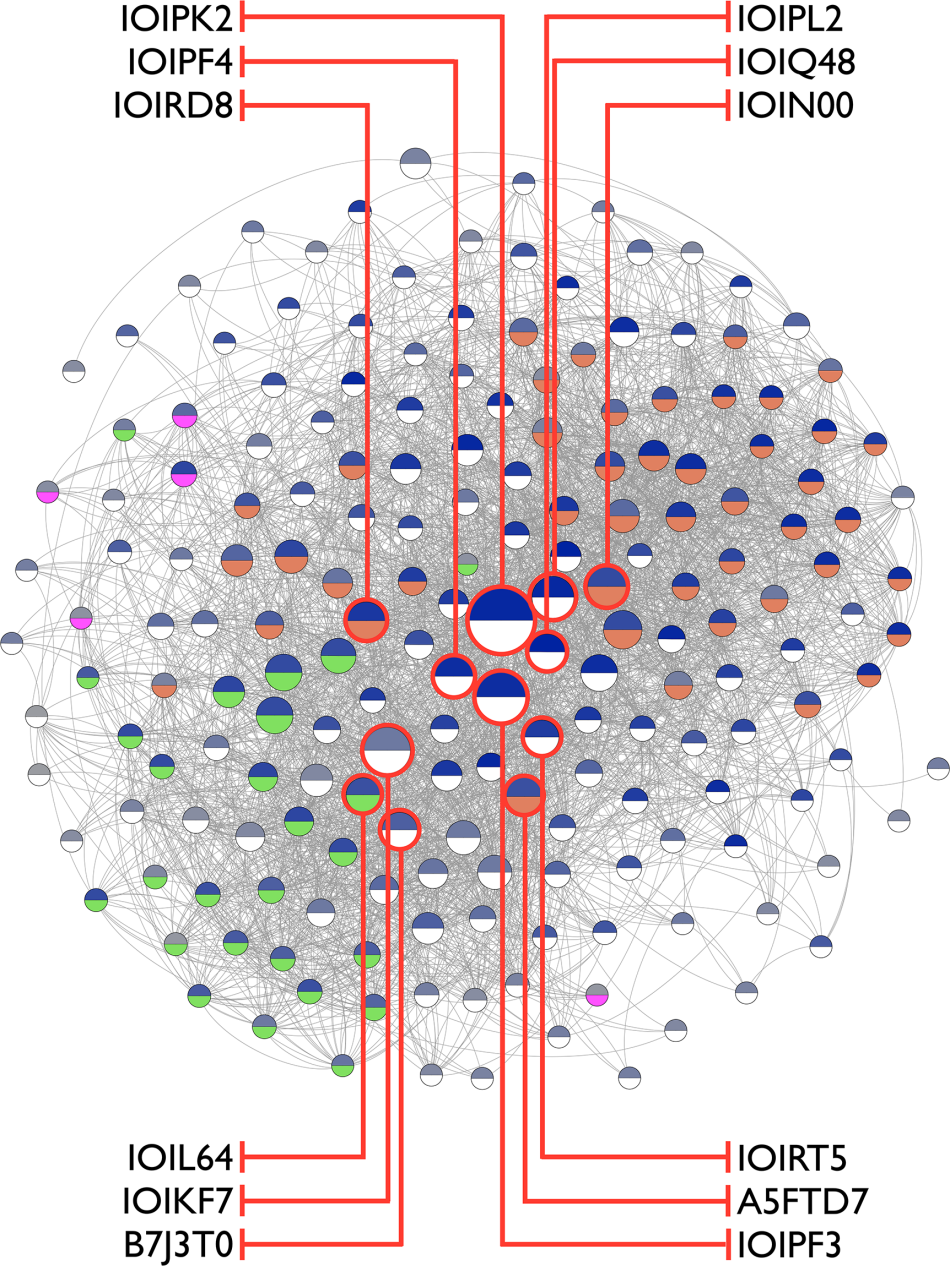
Biofilm ortholog graph. Nodes represent protein coding genes and edges resemble aggregate interaction information. The layout reflects node degree, the node diameter scales with stress centrality. Node color-coding indicates microbial species coverage scaling from one to 25 (upper semicircle, scaling from light to dark blue), and selected COG term assignment in the context of biofilm formation (lower semicircle, orange: cell wall/membrane/envelop biogenesis; green: cell motility; magenta: extracellular structures). Node annotation refers to biomarker candidates according to Table 4.

Ortholog information allows utilizing interaction information across species to derive an ortholog graph, thereby complementing interaction data by aggregation. This procedure enables inclusion of interaction information for finally 191 unique molecular features of the biofilm gene set (S3 Table) in a single network component. 24 proteins lack direct or orthology-derived interaction data and could therefore not be included in the interaction network. The set of 191 protein-coding genes is found to be connected via 6,878 interactions (Fig 2).

The ortholog network allows capturing molecular function of species as such not included in a species set. Representative for such an organism is *L*. *ferrodiazotrophum*, a diazotrophic mesophilic bacterium also found in AMD biofilms [30]. *L*. *ferrodiazotrophum* efficiently fixes nitrogen, but its genome lacks annotation including COG term assignment and interaction data in STRING. The nitrogenase cassette is highly homolog to features found in other diazotrophic bacteria such as *L*. *ferrooxidans* and *Acidithiobacillus* ssp., i.e. the cassette is considered at the ortholog level.

The ortholog graph serves as basis for identifying biomarker candidates according to network topology. Topological parameters characterizing the centrality of a given node capture properties related to the functional importance within its network context. Specifically, node degree and stress centrality resemble properties prone to biomarker candidate selection. Both centrality measures reflect affectedness of state changes in molecular processes, hence serving as proxies for molecular process status. Nodes with high degree centrality establish a large number of interactions, i.e. serve as network hubs [31]. Nodes exhibiting high stress centrality are deemed to be crucially involved in information flow [32]. An analog correlation of degree centrality and influence of a given species in an ecological network was observed for keystone species [33].

The relevance of degree and stress becomes apparent from Fig 2, where high degree is reflected by the central position of respective nodes.

Accordingly, biomarker candidates from the ortholog graph of biofilm formation showing high degree and stress centrality are deemed to serve as relevant proxy for the entire process, and promise sensitivity in monitoring the presence of biofilm formation.

### Candidate biomarker selection

Relevance of network topology aspects is evaluated using proteomics profiles published by Belnap et al., contrasting a generic and an optimized laboratory setting with environmental samples in focus of biofilm formation efficiency [14]. Proteomics comparison of wild type and laboratory cultures provides abundance information for all 191 protein coding genes assigned to biofilm formation. For assessing principal relevance of features included in the biofilm data set, the abundance of AMD (biofilm positive) and generic laboratory culture (“control”) was compared. 25 out of the 191 features are identified as significantly different in abundance. Compared with the background set of microbial genes considered in the experimental study (with less than 5% of all features showing significant differential abundance) proteins assigned to the biofilm formation context see significant overrepresentation with more than 15% of features.

**Fig 3:**
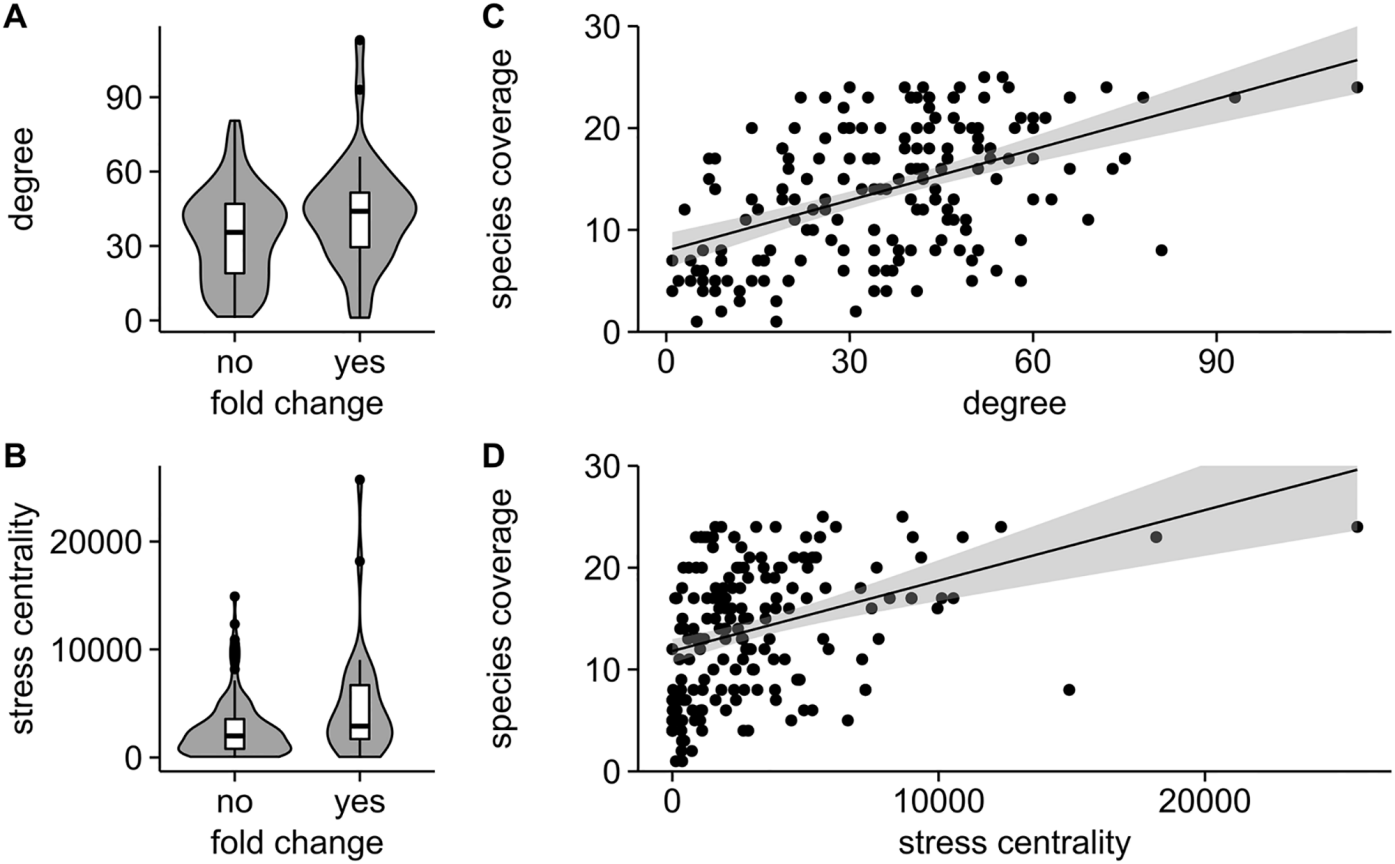
Node details of the biofilm process network in relation to experimental data. Violin plots for (**A**) degree and (**B**) stress centrality of network nodes comparing the wild type microbial community situation with laboratory conditions on fold change significance, complemented by correlation of species coverage with (**C**) degree and (**D**) stress centrality for nodes holding a significant fold change.

Annotation of nodes of the ortholog graph with fold change values assigned to biofilm formation in combination with computation of the topological parameters degree as well as stress centrality allow determining significance in difference of graph measure distributions. Degree as well as stress centrality are found to be different for nodes holding a significant fold change compared to nodes not found to be affected according to the proteomics data (p = 0.038 for degree and p = 0.026 for stress centrality, Fig 3A and 3B). Computing a logistic regression to predict significance in fold change using graph measures as parameters identifies significant individual association (p = 0.022 for degree, p = 0.004 for stress centrality). Aside apparent variance, higher degree and stress centrality appear indicative for significance as well as magnitude of fold change according to the given proteomics data.

A second correlation is determined for graph measures and node species coverage, i.e. evaluating if nodes showing hub properties in degree and stress resemble broad orthology or if they are captured only by a limited set of species. For both parameters a significant (p<0.001) positive correlation is identified (Fig 3C and 3D), aside large variability and sparse data coverage at high values of degree and stress centrality being indicative for positive association.

Based on the observed association of degree and stress centrality with a probability of abundance change under varying biofilm conditions, a rank score of both graph measures and species coverage is determined. Each individual parameter is ranked starting with the maximum value, with the rank score reflecting the mean of the sum of each individual parameter rank for a given protein. Table 4 lists the 12 top ranking proteins (see S3 Table for ranking of all 191 molecular features included in the network).

**Table 4:** Biomarker candidate panel for capturing the biofilm molecular process.

protein name	UniProt ID	prot	Trans	control	# species	function context
**chaperonin GroEL** *(O*: *Post-translational modification*, *protein turnover*, *chaperone functions)*	I0IPK2	+0.6	+2.6	-0.19	24	regulation of protein fate and transport, thermal stability, protection from oxidative damage caused by copper
**chaperone DnaK** *(O*: *Post-translational modification*, *protein turnover*, *chaperone functions)*	I0IPF3	+1.0	+3.0*	-0.08	23	DNA stability, defense against reactive oxygen species
**GrpE protein, putative** *(O*: *Post-translational modification*, *protein turnover*, *chaperone functions)*	I0IPF4	+2.8	+3.0*	-0.35	23	mediation of acid tolerance
**dimethyladenosine transferase** *(J*: *Translation)*	I0IQ48		+2.7		24	quorum sensing: environmental adaptation through cross-species synchronization
**UDP-glucose 4-epimerase, putative** *(M*: *Cell wall/membrane/envelop biogenesis)*	I0IN00			-0.01	17	EPS biosynthesis: adhesion, production of EPS precursors
**diguanylate cyclase, putative** *(T*: *Signal transduction)*	I0IKF7		+2.0	-0.4	8	regulation of bacterial biofilm formation and motility
**diguanylate cyclase/phosphodiesterase** *(M*: *Cell wall/membrane/envelop biogenesis)*	A5FTD7			+0.43	16	EPS biosynthesis: signal transduction
**peptidoglycan glycosyltransferase, putative** *(D*: *Cell cycle control and mitosis; M*: *Cell wall/membrane/envelop biogenesis)*	I0IRD8		+2.0		21	EPS biosynthesis: cell wall modification
**signal transduction histidine kinase** *(N*: *Cell motility; T*: *Signal transduction)*	I0IL64	+6.2	+2.8	-0.33	16	two-component system: chemotaxis regulation
**chaperone clpB** *(O*: *Post-translational modification*, *protein turnover*, *chaperone functions)*	I0IRT5		+7.0	-0.05	20	regulation of stress-response, temperature adaptation: membrane fluidity, pH tolerance
**signal recognition particle subunit FFH/SRP54** *(U*: *Intracellular trafficking and secretion)*	I0IPL2		+2.8	+0.13	25	chemotaxis, acid tolerance, secretion of EPS material
**sensor protein PilS, putative** *(T*: *Signal transduction)*	B7J3T0			-0.28	13	motility: regulation of tight adherence, auto-aggregation, and pili formation

Given are protein name and respective COG term assignment, UniProt database identifier, fold change (optimized vs. standard laboratory culture) in the proteomics data set (prot), fold change (biofilm vs. planktonic fraction) in the transcriptomics data set (trans), fold change (continuous vs. bioleaching culture) in the control data set (control), number of species coding the protein (in the set of 25 species), and protein molecular function context. EPS: extracellular polymeric substance.

### Biomarker evaluation in public domain omics profiles

For evaluation of the biomarker candidate panel two omics studies appear particularly useful due to providing i) substantial coverage of the entire molecular feature space represented in the biofilm network, and ii) showing differential readout for biofilm formation under varying conditions. The first study conducted by Moreno-Paz et al. resembles a prospecting situation and reports transcriptomics profiling comparing planktonic versus biofilm associated bacterial consortia in a natural AMD site [13]. The study confirms significant differential regulation of nine features included in the biomarker panel, all identified as up-regulated in the biofilm context. Although the transcript profile was specifically derived for *L*. *ferrooxidans*, significant orthology of sequences selected in the biomarker panel render fold changes as indicative for biomarker expression on the background of the ortholog network.

A monitoring/process optimization situation can be approximated via comparing proteomics data of the generic and the optimized laboratory condition as provided in Belnap et al. [14]. With optimized conditions designed to reduce metabolic stress AMD communities showed metabolic profiles closer to those of the natural isolates, including an increased capacity in biofilm formation. Four molecular features among the 12 top-ranked candidates are identified as being up regulated in the optimized laboratory setting.

The third data set prepared by Christel et al. compared gene expression of a continuous and a bioleaching (addition of chalcopyrite) culture of *L*. *ferrooxidans* cultivated in an agitated-tank bioreactor [27]. This species is a dominant member of bioleaching consortia (also being involved in consortia studied by Belnap et al. [14] and Moreno-Paz et al. [13]), and is a key driver of biofilm formation. Genes associated with mineral dissolving activity (heavy metal resistance, motility or chemotaxis) were found to be significantly up-regulated under chalcopyrite addition. As expected by the experimental setup, which did not specifically harvest and analyze cells from biofilms, genes assigned to biofilm formation are reported as essentially unchanged in expression. Further, none of the 12 candidate biomarkers are found differently expressed, providing an indication on specificity of candidate markers.

A further data set provided by Vera et al. implemented shotgun proteomics for characterizing early biofilm formation of *A*. *ferrooxidans* on pyrite [34]. In this single organism setting a biofilm monolayer is formed, being essential for cell attachment and subsequent metal sulfide leaching. The early stage biofilm situation is characterized by 87 proteins exhibiting differential abundance, mainly centered around metabolic adoptions. However, only 11 of the 87 proteins are part of the biofilm ortholog graph. This finding may reflect differences when comparing early stage biofilm formation and established biofilms. Even more important, this setting may be indicative for substantial differences of single species biofilm formation and multi-species biofilm scenarios resembling higher order function.

The most prominent function category involved in the biomarker set is chaperones with four protein-coding genes. High ranking in degree centrality reflects broad involvement in folding and arrangement of macromolecular structures as well as their propensity to associate with hub proteins. This property meets with the processes of biofilm formation, but questions specificity, as activity of these proteins is supposedly relevant in a variety of molecular processes beyond the aggregate function in focus.

Still, given chaperones hold specific evidence in the biofilm context in an acidic environment. GroEL chaperones are generally known as stress response proteins and are frequently found in acidophilic bacteria (equivalently identified for the chaperone DnaK) when grown on elementary sulfur. A more specific role of the protein family is modulation of fatty acid components of bacterial cell walls deemed essential during biofilm maturation [35]. The putative GrpE protein as well as the chaperone clpB are described in stress response in acidic environmental conditions and are implicated in the maintenance of acidophilic biofilms [13]. Tsuboi et al. reported meta-transcriptomics data from multiple sampling in an acidic stream ecosystem, identifying GroEL transcripts in a range of physico-chemical conditions (varying temperature and iron concentration) [36]. The second marker, the signal recognition particle subunit FFH/SRP54 (I0IPL2) showed abundance only at distant sites from the spring (characterized by decrease in temperature, iron concentration, and increase in pH), eventually indicative for biofilm formation at these conditions. I0IPL2 was also identified by Peng et al. executing a genomic and transcriptomic analysis of a high altitude AMD community contrasting different temperature conditions [37].

A second prominent function term covers central biofilm components involved in the synthesis of extracellular polymeric substances. Members include a uridine diphosphate (UDP)-glucose 4-epimerase converting uridine diphosphate galactose to UDP-glucose, a precursor for the osmoprotectant trehalose, as well as several exopolysaccharides [38, 39]. Further members include diguanylate cyclase/phosphodiesterase providing cyclic di-guanosine monophosphate (c-di-GMP) as messenger relevant in biofilm formation, complemented by a peptidoglycan glycosyltransferase involved in cell wall modification with respect to biofilm attachment [40].

The third term set covers chemotaxis, sensing and motility, of apparent relevance in structuring the community microenvironment in a biofilm. For dimethyladenosine transferase a more specific function in quorum sensing was discovered and involves environmental adaptation through cross-species synchronization [41]. As second sensing element, srp54 targets secretory proteins to plasma membranes. The signal recognition particle pathway is broadly conserved for targeting polypeptides for secretion. Diguanylate cyclases (as well as phosphodiesterases) are constituents of the bacterial c-di-GMP pathway, and are relevant mediators of bacterial biofilm formation and motility specifically discussed in bioleaching [42]. The sensor protein PilS is involved in a signal transduction system for allowing response to environmental changes, including auto-aggregation of microbial cells involving a signal transduction histidine kinase [43].

Taken together, the panel members represent core functional elements required in establishing a biofilm microenvironment, covering chemotaxis, motility, quorum sensing as well as the production of extracellular matrix components. The prominent representation of chaperones of the GroEL-Dnak-GrpE system not only points to adaptation processes allowing communities to establish in the extreme environments, but also reflects the need for a proper orchestration of the biofilm formation processes [44]. Genes facilitating the quorum sensing function may play an important role in sustaining cooperation of the biofilm community e.g. by fine-tuning its population density [45]. Chen et al. executed a comparative metagenomic and metatranscriptomic analysis of microbial communities in acid mine drainage [46]. The study confirmed presence of four biomarker candidates from Table 4 (chaperonin GroEL, chaperone DnaK, peptidoglycan glycosyltransferase and signal transduction histidine kinase) in all 4 AMD sites analyzed.

Still, the exposed network topology of the panel members being hubs and connectors and on top being encoded in a wide range of species, in various instances covering more than 20 of the in total 25 microbial species involved, may in part be driven by annotation biases of network nodes and interactions, at present not allowing for evidence-based correction. Aside this aspect the need for specialist species for driving aggregate molecular processes is to be addressed. Analyzing species coverage of nodes in the network neighborhood of degree and stress centrality hubs sees more sparse species coverage, indicating the need for marginal consortia members on the molecular process level [47]. As example serves chaperonin GroEL commonly associated with stress response. The protein sees interactions with biofilm-relevant proteins such as a pilus chaperone protein (UniProt ID B7J3A8) involved in surface adhesion, a methyl-accepting chemotaxis sensory transducer (UniProt ID I0IKF9) regulating colony building, a cellulose synthase I (UniProt ID C1F830), together with a pilin signal sequence domain protein (UniProt ID B7J7E7) involved in extracellular polymer production. In the given species set, all such proteins see minor coverage of two to five species. Thus, the ubiquitous GroEL may trigger matrix production through recruitment of specialized function attributed from less abundant species, eventually without seeing significant differential abundance of such contributing factors. Indications are provided in the proteomics study of Belnap et al., where the expression level of any of the matrix synthesis markers (UniProt IDs I0IN00, A5FTD7 and I0IRD8, respectively) remained unchanged across all culturing conditions [14]. These findings are consistent with the reported correlation of high network connectedness with regulatory functionality and a widespread differential expression of regulators and its regulated targets [48].

Molecular features involved in biofilm formation are identified in a range of organisms commonly isolated in AMD and bioleaching sites. For a subset of organisms a sufficient depth of annotation is available both on functional as well as on protein interaction level. Lack of annotation per design removes a subset of protein coding genes from integrative analysis, although eventually resembling excellent biomarker candidates. However, coding regions displaying no orthology to annotated sequences of an extended species set retrieved in a meta-analysis approach may be fairly specific for certain species. In consequence, biomarkers from such subset may lack generalization capacity, in turn limiting sensitivity in a species-agnostic prospecting approach.

Available interaction data allow deriving an ortholog network to approximate the community molecular process of biofilm formation. By integrating orthology information in the network some major limitations, given by unknown or incompletely characterized genes, lack of functional annotation as well as of interaction data can be addressed to some extent. The interaction data source used in this work provides interactions holding experimental evidence and candidate interactions resting on computational inference. With lack of completeness of experimentally derived interactomes together with biases introduced by experimental protocols, inference of interactions from heterogeneous data sources in part addresses given limitations and biases. This approach at the same time introduces probabilistic assignments. In consequence, resulting networks primarily serve hypothesis generation, demanding subsequent experimental evaluation. On the other hand, correlation of abundance of sequences with unknown function and annotated nodes of a mechanistic network model may support candidate function assignment [49].

Combining various functionalities deemed necessary for effective biofilm formation in a multimarker panel promises improved sensitivity as well as specificity. The functional diversity of the process of forming a biofilm microenvironment, specifically beyond enzymes, points to an important aspect for selecting a comprehensive network model. For example, approaches restricted to metabolic networks by e.g. utilizing KEGG orthology (KO) terms of enzymes (nodes) connected by metabolites (edges) cover less than half of the network nodes included in the biofilm orthology graph [11]. In addition, only 5 out of the 12 panel members identified in this study hold enzymatic function.

## Conclusion

The workflow discusses candidate biomarker retrieval for prospecting of an aggregate biological function executed by microbial consortia in environmental samples. The approach leverages on cross-species interaction consolidated as ortholog network, and utilizes topological properties of network nodes for molecular feature selection.

Identification of candidate panel members is based on a molecular process model tailored at representing the molecular context of biofilm formation. The candidate biomarkers are selected according to a rank score of degree and stress centrality as well as species coverage, thus focusing on prevalent proteins being deemed essential for establishing a biofilm microenvironment. Evaluating candidate biomarkers via utilizing public domain omics profiles indicates relevance in biofilm formation. Applicability of the method is not restricted to biofilm prospecting in copper mineral decomposition, but is expandable to alternative microbial community functions including as examples biofertilization in agriculture or microbial communities serving in oil spill bioremediation.

Practical implementation on the mRNA level, e.g. via quantitative polymerase chain reaction (qPCR)-based methods, demands conserved sequence regions as probes. The approach of defining a set of biomarkers designed to screen for a given molecular function being at least to some extent independent of the executing microbes bears the promise to significantly facilitate prospecting of novel bacterial consortia. The precise composition of consortia is in most cases unknown at the point of screening. A species-robust screening tool supersedes the need for any prior knowledge on likely species occurrence. In line with prospecting goals a biomarker panel allows a first evaluation of samples, and in case of positive biomarker readout providing grounds towards a more detailed molecular analysis e.g. via metagenomics. This is particularly useful in cases where potential prospecting areas are large and diverse, or where a fast and frequent read-out, e.g. in a monitoring situation, is needed. Further, the approach facilitates the targeted discovery of new species in a functional context in focus, particularly if combined with novel techniques such as methods for *de novo* functional characterization of unknown species.

In conclusion, interaction networks offer a methodology for characterization of community aggregate molecular functions via integrating individual species contributions into a molecular process network. Next to adding to our understanding of molecular mechanisms of aggregate function such networks allow selecting candidate biomarkers grounded on network topology characteristics. Such biomarkers promise support in microbial function prospecting as well as in industrial process monitoring in a range of application areas, with bioleaching and bioremediation as examples.

## Supporting information

S1 Table Complementary scientific references, data aggregation. Spreadsheet “sites”: Copper site-specific scientific references; spreadsheet “species”: Microbial species-specific scientific references; spreadsheet “functions”: key scientific references covering molecular function terms.
(XLSX)

S2 Table Protein coding genes identified as relevant in copper bioleaching/AMD. Provided is the protein name together with identifiers from database references of UniProt, PATRIC and IMG.
(XLSX)

S3 Table Protein coding genes embedded in the biofilm process network. The listing holds genes assigned to the functional term biofilm formation embedded in the ortholog network, respective topology measures of degree and stress centrality, species coverage, and ranked sum of topological parameters and species coverage.
(XLSX)
